# Acute Myopericarditis Likely Secondary to Disseminated Gonococcal Infection

**DOI:** 10.1155/2015/385126

**Published:** 2015-07-12

**Authors:** Daniel Bunker, Leslie Dubin Kerr

**Affiliations:** Division of Rheumatology, Department of Medicine, Icahn School of Medicine at Mount Sinai, New York, NY 10029, USA

## Abstract

Disseminated gonococcal infection (DGI) is a rare complication of primary infection with *Neisseria gonorrhoeae*. Cardiac involvement in this condition is rare, and is usually limited to endocarditis. However, there are a number of older reports suggestive of direct myocardial involvement. We report a case of a 38-year-old male with HIV who presented with chest pain, pharyngitis, tenosynovitis, and purpuric skin lesions. Transthoracic echocardiogram showed acute biventricular dysfunction. Skin biopsy showed diplococci consistent with disseminated gonococcal infection, and treatment with ceftriaxone improved his symptoms and ejection fraction. Though gonococcal infection was never proven with culture or nucleic acid amplification testing, the clinical picture and histologic findings were highly suggestive of DGI. Clinicians should consider disseminated gonococcal infection when a patient presents with acute myocarditis, especially if there are concurrent skin and joint lesions.

## 1. Introduction

*Neisseria gonorrhoeae* is a Gram-negative diplococcus that has a tropism for superficial human mucosal surfaces, typically causing infection of the urethra, cervix, and pharynx. In approximately 0.5–3% of patients, the bacteria disseminate in the bloodstream [1], but even with dissemination, cardiac involvement is rare. We report a patient with the acute onset of biventricular cardiac dysfunction likely due to disseminated gonococcal infection.

## 2. Report of a Case

**Figure 1:**
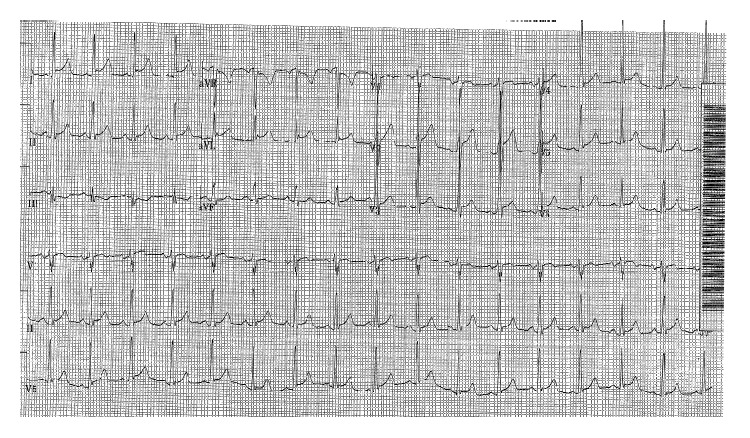
Initial EKG.

A 38-year-old male with a history of HIV and a non-ST elevation myocardial infarction (NSTEMI) was transferred from an outside hospital for the acute onset of biventricular congestive heart failure. Two days prior to presentation, the patient had noted fever and rigors with sore throat and right sided neck swelling. The next day he developed polyarticular joint pain, most prominent in the right ankle, right knee, and bilateral hands, as well as a tender, purpuric rash on his lower extremities. At that point, he began taking amoxicillin, which he had left over from a previous infection. He subsequently developed substernal chest pain which prompted presentation to his local emergency department. On arrival, he was found to be hypotensive with EKG showing diffuse ST elevations (Figure 1). Emergent cardiac catheterization showed no acute coronary abnormality; however, a 2D transthoracic echocardiogram revealed new severely reduced biventricular systolic function with an ejection fraction of 20% (a 2D echo obtained four months prior had shown normal biventricular function). He was transferred to our institution for further management.

**Figure 2:**
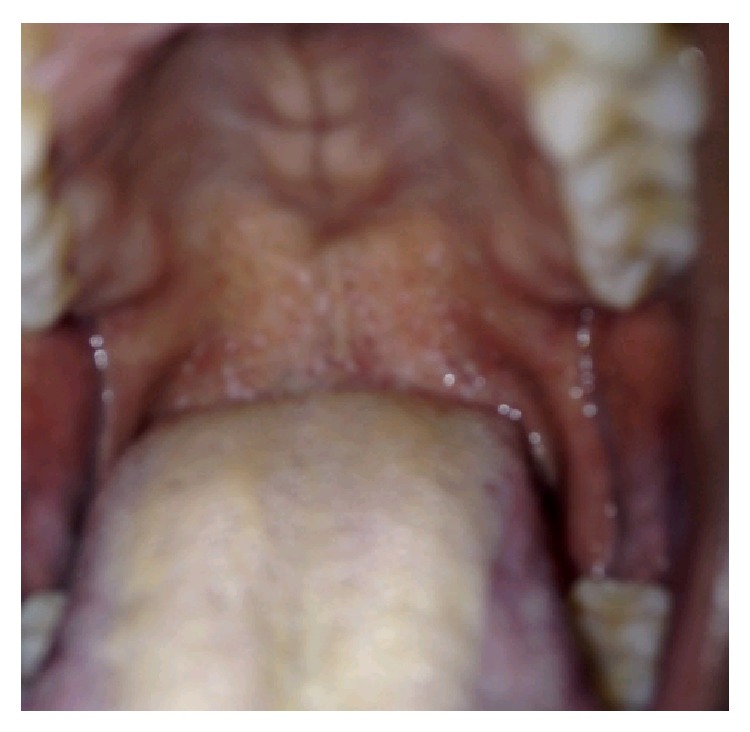
Oropharynx.

**Figure 3:**
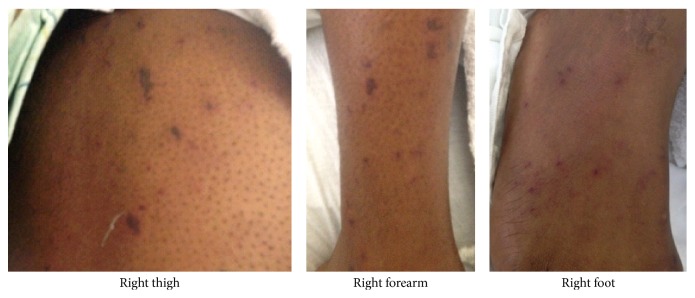
Skin findings.

**Figure 4:**
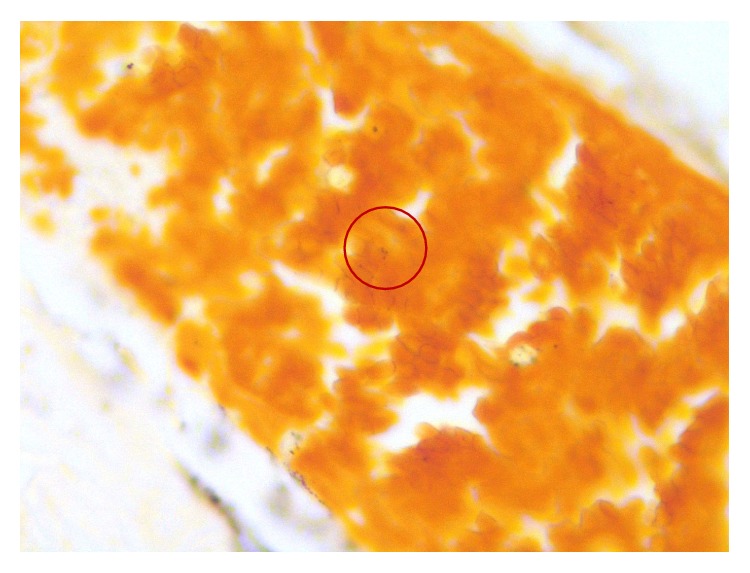
Skin biopsy, Steiner stain.

The patient arrived in the coronary care unit on inotropic support but not in acute distress. He reported good compliance with his HIV medication regimen (emtricitabine, rilpivirine, and tenofovir), with a baseline CD4 count >500 cells/mm^3^ and no history of opportunistic infections. He also had hypertension and had undergone drug-eluting stent placement to his left anterior descending artery for an NSTEMI three years prior. He had sex with men (anal and oral intercourse) and reported two sexual partners over the past month. Examination showed white patches in his posterior pharynx (Figure 2) but no cervical lymphadenopathy. Cardiac exam showed a regular rate and rhythm, with normal heart sounds and no murmurs or rubs. His jugular veins were not distended. He was unable to make a fist with either hand, had minimal range of motion of the wrists and right ankle, and had prominent metacarpophalangeal joint pain with passive flexion and extension of his fingers, indicating tenosynovitis. Skin exam showed multiple small purpuric stellate papules and patches with mildly erythematous borders on his upper and lower extremities (Figure 3). His CRP was elevated to 400 milligrams/liter and his troponin peaked at 19 nanograms/milliliter. He refused myocardial biopsy. However, a skin biopsy revealed extensive intravascular fibrin thrombi with secondary epidermal changes; Gram and Steiner stains showed rare diplococci within the vessels (Figure 4).

The patient was started on intravenous daily ceftriaxone and also received one dose of azithromycin for presumed disseminated gonococcal infection, as per the 2010 guidelines from the Centers for Disease Control. Though blood cultures remained sterile and specific gonococcal cultures including pharyngeal cultures (taken after antibiotics were started) were negative, the patient had rapid improvement with this treatment and within two days was able to ambulate comfortably and write with a pen. His ejection fraction increased to 46% on repeat echocardiogram. After five days of therapy, the patient left against medical advice before contact tracing could be performed.

## 3. Discussion

The incidence of gonorrhea has declined significantly since its peak in the 1970s; however, it remains high among men who have sex with men (MSM). Additionally, antibiotic resistance is growing and is a major public health concern, with fluoroquinolones and oral cephalosporins now no longer recommended as empiric treatment for gonococcal infection [2]. Disseminated gonococcal infection (DGI) typically occurs 2-3 weeks after primary infection [3], and a lack of concurrent urogenital symptoms, as seen in our patient, is common. Given our patient's history of sore throat with cervical lymphadenopathy, evidence of pharyngitis on exam, and report of receptive oral intercourse, primary pharyngeal infection was suspected, which may be a risk factor for dissemination [4]. Although data suggests that gonococcal infection is more likely in MSM who have underlying HIV [5], it is unknown whether the presence of HIV infection is a risk factor for dissemination of gonorrhea once the patient is infected.

Patients with disseminated gonococcal infection typically present with one of two clinical phenotypes: a tenosynovitis-arthritis syndrome with prominent systemic manifestations or an isolated suppurative arthritis usually involving a single large joint. With either phenotype, DGI rarely involves the heart, and when it does, endocarditis [6] and/or isolated pericarditis [7] are the usual manifestations. Cases of myopericarditis are extremely rare and mostly confined to the preantibiotic era [8]. In a classic case series of 42 patients with DGI from 1971, five patients had EKG abnormalities consistent with myocarditis (mostly T wave inversions) that subsequently resolved with treatment [9]. In 1974, Fraser et al. reported a 22-year-old patient with fever, arthritis, skin rashes, and gonococcemia; EKG showed a pulse of 140 with inverted and flattened T waves with multiple ventricular extra systolic beats, consistent with acute myocarditis [10]. More recent case series [11, 12] have not found evidence of myocarditis in patients with DGI. The documentation of acute biventricular dysfunction by echocardiography in this case of suspected DGI is a unique finding.

The diagnosis of disseminated gonococcal infection is made when any evidence of systemic or primary gonococcal infection is found in a patient with compatible symptoms of DGI. Even with the tenosynovitis-dermatitis phenotype as seen in our patient, blood cultures are often negative [13]. Additionally, our patient had taken antibiotics at home prior to presentation, further reducing the culture yield in both the blood and the pharynx. Despite this, small diplococci consistent with* Neisseria gonorrhoeae* were seen in the vasculature on both Gram stain and Steiner stain (a qualitative histologic silver stain very sensitive for bacteria) of the skin biopsy specimen, which is uncommon but can be seen in DGI [14]. As* Neisseria gonorrhoeae* was not cultured nor identified via nucleic acid amplification, we could not be completely certain of the diagnosis. However, in this case, we were able to make the clinical diagnosis of DGI based on the morphologic appearance of diplococci on skin biopsy, prominent tenosynovitis, tender purpuric skin lesions, compatible sexual history, and the rapid resolution of symptoms with appropriately tailored antibiotic therapy.

## 4. Conclusions

This case illustrates a rarely seen but important manifestation of disseminated gonococcal infection. The echocardiographic finding of acute biventricular dysfunction in this case is notable. Clinicians should consider DGI in patients with acute myocarditis, especially if there are concurrent joint and skin findings.
